# Association of anemia and platelet activation with necrotizing enterocolitis with or without sepsis among low birth weight neonates: a case–control study

**DOI:** 10.3389/fped.2023.1172042

**Published:** 2023-08-31

**Authors:** Zhou Jiang, Guangyong Ye, Songying Zhang, Long Zhang

**Affiliations:** ^1^ Sir Run Run Shaw Hospital, Zhejiang University School of Medicine, Hangzhou, China; ^2^ Women’s Hospital, Zhejiang University School of Medicine, Hangzhou, China

**Keywords:** NEC, anemia, sepsis, the value of the proportion of large platelets, platelet crit

## Abstract

**Background:**

This study aims to evaluate the value of the proportion of large platelets (PLCR) and platelet crit (PCT) in predicting necrotizing enterocolitis (NEC) in low birth weight (LBW) neonates.

**Methods:**

A total of 155 LBW (<2,500 g) neonates with NEC, who were admitted to the neonatal intensive care unit (NICU) of the hospital from January 1, 2017, to November 30, 2019, were included in the case group. According to the 1:3 case–control study design, a total of 465 LBW neonates without NEC (three for each LBW neonate with NEC), who were admitted to the NICU and born ≤24 h before or after the birth of the subjects, were included in the control group.

**Results:**

During the study period, a total of 6,946 LBW neonates were born, of which 155 had NEC, including 92 who also had sepsis. Neonatal sepsis was the most important risk factor and confounding factor for NEC in LBW neonates. Further stratified analysis showed that in LBW neonates without sepsis, anemia [*P* = 0.001, odds ratio (OR) = 4.367, 95% confidence interval (CI): 1.853–10.291], high PLCR (*P *< 0.001, OR = 2.222, 95% CI: 1.633–3.023), and high PCT (*P* = 0.024, OR = 1.368, 95% CI: 1.042–1.795) increased the risk of NEC and the receiver operating characteristic curve area of PLCR, sensitivity, specificity, and cutoff value were 0.739, 0.770, 0.610, and 33.55, respectively.

**Conclusions:**

The results showed that 2/100 LBW neonates were at risk for NEC, and the stratified analysis of the confounding factors of sepsis identified the risk factors of NEC in LBW neonates. This study first reported the significance of PLCR in the early prediction of NEC occurrence in LBW neonates without sepsis.

## Introduction

Neonatal necrotizing enterocolitis (NEC) is a neonate-specific inflammatory necrotizing disease that involves the ileum and/or colon and is common among premature infants, severely threatening the life of neonates (1–3). The mortality rate has declined due to continuous advances in the treatment of premature infants in recent years, but the incidence of NEC has not decreased significantly.

Statistically, the incidence of NEC in premature infants weighing <1,500 g was 5%–10%, the mortality rate was 20%–30%, and >30%–50% of NEC neonates required surgical treatment (4, 5). Despite decades of research, the current understanding of the diagnosis and treatment of neonatal NEC is limited, the rate of neonatal NEC mortality remains high, and neonatal surgical advances have not significantly improved the prognosis in NEC survivors (6, 7). Therefore, NEC intervention, especially in low birth weight (LBW) neonates with NEC, should be under intensive focus with respect to identifying the causes and related factors for early diagnosis and treatment.

Hitherto, although NEC pathogenesis remains unclear, several studies have shown that it is caused by a combination of factors. Some studies have reported that preterm birth (6, 7), low birth weight (6–9), and race (8) were critical risk factors. Recent studies have shown that maternal infection (10), congenital pneumonia (11), asphyxia (12), blood transfusion (12), anemia (13, 14), and neonatal sepsis are also potential contributing factors. Furthermore, it has been suggested that NEC pathogenesis is multifactorial, involving a combination of abnormal bacterial colonization, a cascade amplification of inflammation, gut immaturity, and ischemia–reperfusion (I/R) injury. The differences in the immune response to mucosal damage and the microbiota may also be responsible for the increased inflammatory response in NEC (15). NEC induced by gut immaturity and I/R injury is not significantly associated with sepsis. Based on the epidemiological and clinical theories, sepsis can confound the diagnosis of clinical complications and the use of inflammatory proteins as the NEC marker. Both sepsis and NEC require careful differential diagnosis, as both may be lethal if not properly diagnosed and treated.

Regarding gut immaturity in NEC infants, the degree and duration of thrombocytopenia are associated with the severity of bowel injury and adverse clinical outcomes. NEC infants develop thrombocytopenia with a platelet (PLT) count of <100 × 10^9^/L. This low PLT count is yet an unresolved clinical dilemma (16, 17). A robust NEC biomarker different from that of sepsis could improve bedside management, reduce morbidity and mortality rates, and allow patients to select potential treatments in the clinic. Animal studies (18) have shown that PLT activation during NEC-like intestinal injury is an early, thrombin-mediated process that antedates both mucosal damage and the rise in bacterial products in plasma. Hence, it is a crucial pathophysiological event during neonatal intestinal injury. In clinical practice, the PLT count in NEC patients is monitored periodically, but PLT activation indicators, such as the mean platelet volume (MPV), platelet crit (PCT), platelet distribution width (PDW), and platelet large cell ratio (PLCR), are usually neglected (19, 20). These PLT indicators are valuable in the clinical diagnosis and prognostic prediction of cardiovascular and metabolic diseases (21, 22); however, their clinical significance, reference value, and utilization value are still being investigated.

Intriguingly, the high mortality rate of NEC patients could be ascribed to the difficulty in diagnosing and treating the condition promptly. Radiographic evidence, such as pneumatosis intestinalis, is used to diagnose severe or advanced disease but has a sensitivity of only 44% with limited specificity and a lack of interpretation concordance (2, 23). Several studies have adopted 1:1 or 1:2 case–control cohorts with low statistical efficiency (24, 25). Herein, we hypothesized that sepsis, anemia, and PLT activation index are vital NEC predictors in LBW neonates. Furthermore, a 1:3 case–control study with sufficient statistical efficiency was conducted to verify the predictive value of anemia, sepsis, and PLT activation indexes in the incidence of LBW NEC, to achieve earlier diagnosis and treatment.

## Methods

### Participants

A total of 155 LBW (<2,500 g) neonates with NEC, who are born in the Women's Hospital, Zhejiang University School of Medicine, Hangzhou, China, and admitted to the neonatal intensive care unit (NICU) of the hospital from January 1, 2017, to November 30, 2019, were included in the case group. According to the 1:3 case–control study design, a total of 465 LBW neonates without NEC (three for each LBW neonate with NEC), who are admitted to the NICU and born ≤24 h before or after the birth of the subjects, were included in the control group.

The exclusion criteria for the case and control groups were as follows: neonates who were unlikely to survive or had significant gastrointestinal anomalies or those who were discharged at their own will in 3 days. Among the neonates excluded in our study, none with severe abdominal distension was suspected of having fulminant NEC.

All patient information was obtained from the hospital medical record database.

Neonates were diagnosed with NEC (stage II or above) if they met the diagnostic standard in the Practical Neonatology and Bell staging (26, 27) and had clinical symptoms such as abdominal distension, vomiting, and bloody stool triad and if the abdominal plain x-ray scan revealed abdominal intestinal aeration, intestinal obstruction, intestinal pneumatosis, or intrahepatic portal venous gas.

Sepsis in infants is defined by the presence of signs and symptoms of infection with positive blood culture. It is classified as early-onset sepsis (EOS) if symptoms start before 72 h of life and late-onset sepsis (LOS) if symptoms start after 72 h of life. NEC with sepsis is diagnosed in infants with signs and symptoms of infection and positive blood cultures before NEC.

The hospital routinely monitors the complete blood count (CBC) of high-risk infants with stable condition weekly, and the frequency of examination will be increased accordingly for infants with unstable condition. Neonatal anemia is defined as Hb levels less than the fifth percentile, with Hb levels varying with gestational age (28).

### Identification of factors

The main influencing factors of the included subjects were listed, and clinical information of the subjects, including maternal factors [age, nationality, number of fetuses, hypertensive disorders complicating pregnancy (HDCP), gestational diabetes mellitus (GDM), placenta previa (PP), placenta abruption, and premature rupture of membrane (PROM)] and neonatal factors [date of birth, birth weight, NEC diagnosis age (the timespan from the date of birth to NEC diagnosis), neonatal sepsis, patent ductus arteriosus (PDA), anemia, hypoglycemia, birth asphyxia (29), blood transfusion, mycoplasma infection (30), and hyperglycemia], were obtained from the hospital medical record database every 3 months. The complications in the subjects occurred before NEC diagnosis. PLT indicators (such as PLT count, PDW, MPV, PCT, and PLCR) of the case group were recorded as the latest data 2 days before the date of NEC diagnosis, whereas those of the control group were recorded 1 day before and after the date of NEC diagnosis of the subjects.

### Assignment of main study variables

#### Dependent variables

NEC neonates were assigned 1, and the controls were assigned 0.

#### Independent variables

All independent variables (quantitative or qualitative) were converted to qualitative variables by assignment. Since there were no definitive clinical reference values for PLT indicators for neonates, especially for neonates with LBW or low gestational age, the independent variables were stratified and assigned values using the quartile (Q) method, namely, quartile 1 (Q1), quartile 2 (Q2), and quartile 3 (Q3) (Table 1).

**Table 1:** Assignments of independent variables.

Variable		Assignment	Variable		Assignment
Maternal factor			Neonatal factor		
Gestational weeks	≤28	0	Gender	Male	0
	29–37	1		Female	1
	≥37	2	Birth weight (g)	<1,000	0
Number of fetuses	Single fetus	0		1,000–1,500	1
	Multiple fetuses	1		1,500–2,500	2
HDCP	No	0	Transfusion	No	0
	Yes	1		Yes	1
GDM	No	0	Sepsis	No	0
	Yes	1		EOS	1
				LOS	2
PP	No	0	PDA	No	0
	Yes	1		Yes	1
Placenta abruption	No	0	Anemia	No	0
	Yes	1		Yes	1
PROM	No	0	Hypoglycemia	No	0
	Yes	1		Yes	1
			Asphyxia	No	0
				Yes	1
			Mycoplasma infection	No	0
				Yes	1
			Hyperglycemia	No	0
				Yes	1
			PLT	<Q1: 0; Q1–Q2: 1; Q2–Q3: 2; ≥Q3: 3;	
			PDW	<Q1: 0; Q1–Q2: 1; Q2–Q3: 2; ≥Q3: 3;	
			MPV	<Q1: 0; Q1–Q2: 1; Q2–Q3: 2; ≥Q3: 3;	
			PCT	<Q1:0; Q1–Q2:1; Q2–Q3:2; ≥Q3:3;	
			PLCR	<Q1: 0; Q1–Q2: 1; Q2–Q3: 2; ≥Q3: 3	

### Statistical analysis

The general characteristics were compared between the case and control groups. The birth weight of the NEC neonates was determined to be normally distributed by the Shapiro–Wilk test, and Student’s *t*-test was used to compare the birth weight of the NEC neonates with and without sepsis. Spearman's method was used for the analysis of gestational age, birth weight, and NEC diagnostic age. In the 1:3 case–control analysis, the occurrence of NEC was evaluated by analyzing the variables using univariate non-conditional logistic regression. A factor with *P* < 0.5 was analyzed using stepwise multivariate conditional logistic regression (Cox regression). The factors, after being stratified by sepsis, were analyzed using univariate and multivariate non-conditional logistic regression. The receiver operating characteristic (ROC) curve was used to evaluate the PLCR and PCT in NEC diagnosis. SPSS 22.0 software was used for statistical analyses and image drawing. Data conforming to the normal distribution were expressed as mean ± standard deviations, while those not conforming to the normal distribution were expressed as median (minimum, maximum). The odds ratio (OR) and the 95% confidence interval (CI) were calculated. An OR with 95% CI not containing the value of 1 was of statistical significance. All tests were two-sided, and *P* < 0.05 was considered statistically significant.

## Results

### Results of the general information

**Figure 1:**
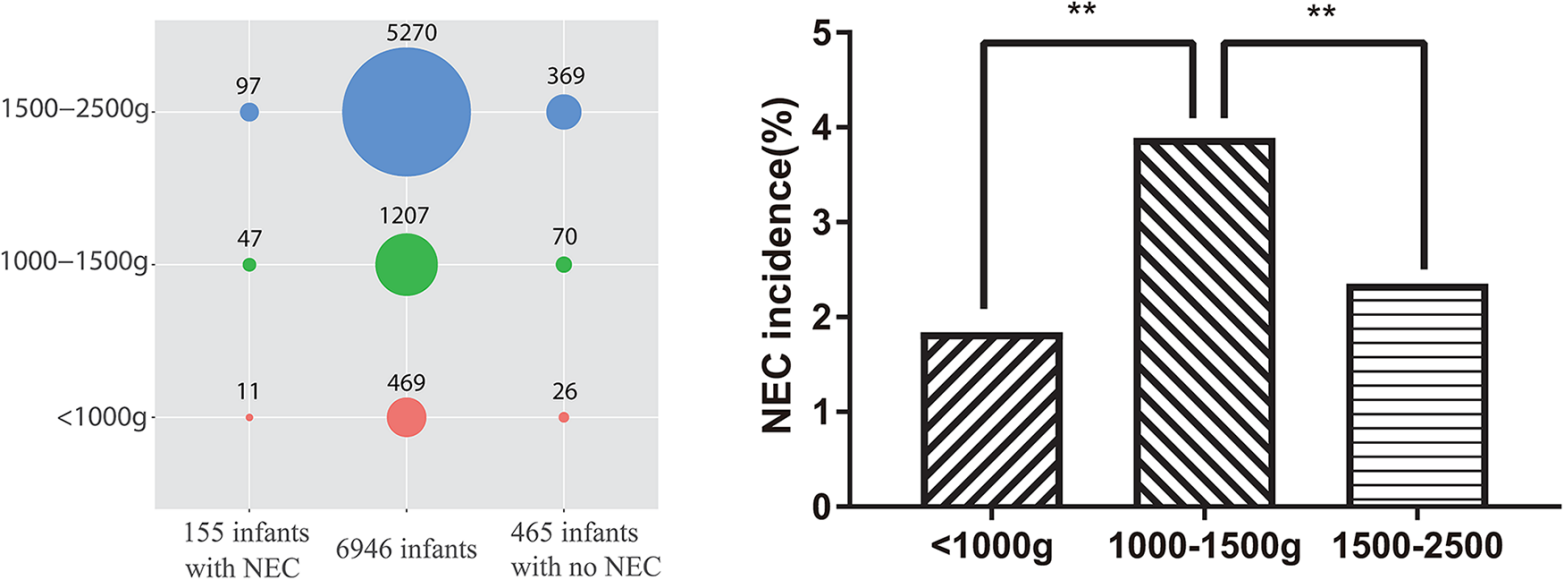
Diagnoses of enrolled infants in the 1:3 case–control study.

During the study period, a total of 58,507 mothers gave birth to a total of 60,182 neonates in the hospital. Among the neonates, 6,946 (11.54%) had LBW, and 155 (2.23%) had LBW and NEC [including 92 (59.35%) who also had sepsis]. Among the 465 neonate controls with LBW in this study, 58 (12.47%) also had sepsis. Among the 6,946 LBW neonates, 5,270 (75.87%) had a birth weight between 1,500 and 2,500 g [including 97 (1.84%) with NEC], 1,207 (17.38%) had a birth weight between 1,000 and 1,500 g [including 47 (3.89%) with NEC], and 469 (6.75%) had a birth weight of <1,000 g [including 11 (2.35%) with NEC], which are evaluated using the chi-square test, as shown in Figure 1. Moreover, one of the NEC neonates had a mother with Lahu nationality, and the others had mothers with Han nationality. There was no statistically significant difference between the case and control groups in gestational age, *P* > 0.05. The general information on LBW neonates and their mothers is shown in Table 2.

**Table 2:** General information on LBW neonates and their mothers in the case–control study.

Factors		Case group (*n* = 155)	Control group (*n* = 465)
Maternal factor			
Age (years)		31.35 ± 4.59	31.10 ± 4.51
Nationality (*n*)	Han Chinese	154	465
	Minority	1	0
Gestational week [median (minimum, maximum)]		31 (21, 39)	34 (24, 39)
Number of fetuses (*n*)	Single fetus	104	288
	Multiple fetuses	51	177
HDCP (*n*)		18	55
GDM (*n*)		27	72
PP (*n*)		18	51
Placenta abruption (*n*)		20	23
PROM (*n*)		49	131
Neonatal factor			
Gender (*n*)	Male	86	226
	Female	69	239
Blood transfusion (*n*)		18	62
Birth weight (g)		1,588.32 ± 384.70	1,878.93 ± 449.60
Sepsis (*n*)		92	58
EOS		22	36
LOS		70	22
PDA (*n*)		34	82
Anemia (*n*)		21	20
Hypoglycemia (*n*)		15	51
Asphyxia (*n*)		22	68
Mycoplasma infection (*n*)		11	16
Hyperglycemia (*n*)		11	10
PLT (10^9^/L)		321.73 ± 105.60	310.32 ± 110.62
PDW (fl)		14.93 ± 2.92	13.17 ± 3.02
MPV (fl)		11.77 ± 0.87	11.00 ± 1.07
PCT (%)		0.38 ± 0.11	0.34 ± 0.12
PLCR (%)		37.77 ± 6.60	31.89 ± 8.13

### Univariate analysis between NEC occurrence and baseline characteristics and clinical information of mothers

The cause of NEC is complex and might be affected by multiple maternal factors (such as general characteristics and complications). The univariate analysis of the main complications revealed that the gestational week and placenta abruption were statistically correlated (Table 3). Placenta abruption (OR = 2.847, 95% CI: 1.517–5.343, *P* = 0.001) was identified as a risk factor for NEC occurrence, whereas gestational week (OR = 0.321, 95% CI: 0.201–0.512, *P* < 0.001) was the independent protective factor.

**Table 3:** Univariate analysis between NEC occurrence and baseline characteristics and clinical information of mothers.

	*B*	SE	*P*	OR	95% CI	
					Lower limit	Upper limit
Gestational week	−0.272	0.036	<0.001	0.321	0.201	0.512
HDCP	−0.021	0.289	0.943	0.979	0.556	1.725
GDM	0.141	0.248	0.569	1.151	0.709	1.870
PP	0.064	0.291	0.825	1.067	0.603	1.888
Multiple fetuses	−0.226	0.196	0.249	0.798	0.544	1.171
Placenta abruption	1.046	0.321	0.001	2.847	1.517	5.343
PROM	0.16	0.201	0.414	1.18	0.800	1.750

### Univariate analysis between NEC occurrence and baseline characteristics and clinical information of LBW neonates

The literature showed that a correlation has been in the spotlight between NEC occurrence and baseline characteristics and complications in neonates. The univariate analysis revealed that in neonates, sepsis, PDA, anemia, hyperglycemia, and birth weight were statistically correlated. Sepsis (*P* < 0.001, OR = 10.247, 95% CI: 06.717–15.633), PDA (*P* = 0.018, OR = 1.680, 95% CI: 1.094–2.580), anemia (*P* < 0.001, OR = 3.482, 95% CI: 1.835–6.627), and hyperglycemia (*P* = 0.005, OR = 3.476, 95% CI: 1.447–8.351) were identified as risk factors for NEC occurrence, whereas gestational week (OR = 0.606, 95% CI: 0.453–0.812, *P* = 0.001) was the independent protective factor. Conversely, hypoglycemia (OR = 0.870, 95% CI: 0.474–1.596, *P* = 0.651), asphyxia (OR = 0.966, 95% CI: 0.575–1.623, *P* = 0.895), wet lung (31) (OR = 1.483, 95% CI: 0.986–2.231, *P* = 0.058), mycoplasma infection (OR = 2.144, 95% CI: 0.973–4.725, *P* = 0.059), and blood transfusion (OR = 0.854, 95% CI: 0.488–2.231, *P* = 0.580) were not statistically correlated with NEC occurrence in LBW neonates. In addition, the analysis of the correlation between NEC occurrence and PLT indicators in LBW neonates showed that the PLT count (OR = 1.494, 95% CI: 0.953–1.322, *P* = 0.167) was not statistically correlated, whereas PDW (OR = 1.920, 95% CI: 1.597–2.307, *P *< 0.001), MPV (OR = 2.093, 95% CI: 1.735–2.525, *P *< 0.001), PCT (OR = 1.441, 95% CI: 1.217–1.705, *P *< 0.001), and PLCR (OR = 2.156, 95% CI: 1.777–2.017, *P *< 0.001) were statistically correlated with NEC occurrence in LBW neonates (Table 4).

**Table 4:** Univariate analysis between NEC occurrence and baseline characteristics and clinical information of LBW neonates.

	*B*	SE	*P*	OR	95% CI	
					Lower limit	Upper limit
Sepsis	2.327	0.216	<0.001	10.247	6.717	15.633
PDA	0.519	0.219	0.018	1.680	1.094	2.580
Anemia	1.249	0.328	<0.001	3.482	1.835	6.627
Hypoglycemia	−0.140	0.310	0.652	0.870	0.474	1.596
Asphyxia	−0.035	0.265	0.895	0.966	0.575	1.623
Wet lung	0.394	0.208	0.058	1.483	0.986	2.231
Mycoplasma infection	0.763	0.403	0.059	2.144	0.973	4.725
Hyperglycemia	1.246	0.447	0.005	3.476	1.447	8.351
Blood transfusion	−0.158	0.285	0.580	0.854	0.488	1.494
Birth weight	−0.500	0.149	0.001	0.606	0.453	0.812
PLT	0.115	0.084	0.167	1.122	0.953	1.322
PDW	0.652	0.094	<0.001	1.920	1.597	2.307
MPV	0.739	0.096	<0.001	2.093	1.735	2.525
PCT	0.365	0.086	<0.001	1.441	1.217	1.705
PLCR	0.768	0.099	<0.001	2.156	1.777	2.017

### Analysis of the effect of multiple factors and their correlation on NEC occurrence

To avoid missing the critical clinical factors, we considered 16 variables with *P *< 0.05 in the univariate regression analysis result as independent variables for the 1:3 case–control analysis using the stepwise multivariate conditional logistic regression (the survival function was assessed by Cox regression). The results showed that sepsis, PLCR, and PCT were the final factors in the regression model. EOS (OR = 2.424, 95% CI: 1.461–4.021, *P* = 0.001) and LOS (OR = 4.291, 95% CI: 3.001–6.138, *P *< 0.001) increased the risk of NEC in LBW neonates. Increased PLCR (OR = 1.451, 95% CI: 1.220–1.724, *P *< 0.001) and increased PCT (OR = 1.225, 95% CI: 1.056–1.422, *P* = 0.007) could be the risk factors for NEC occurrence, as shown in Table 5.

**Table 5:** Multivariate logistic regression (survival function Cox regression) analysis of NEC occurrence in LBW neonates.

	*B*	SE	*P*	OR	95% CI	
					Lower limit	Upper limit
Sepsis	1.285	0.170	<0.001	3.614	2.589	5.046
EOS	0.885	0.258	0.001	2.424	1.461	4.021
LOS	1.457	0.183	<0.001	4.291	3.001	6.138
Anemia	0.516	0.237	0.030	1.675	1.053	2.665
PLCR	0.372	0.088	<0.001	1.451	1.220	1.724
PCT	0.203	0.076	0.007	1.225	1.056	1.422

### Analysis of the effect of multiple factors and their correlation on NEC occurrence in neonates without sepsis

The effect of sepsis weighted the most among the above factors of statistical significance. Sepsis could lead to systemic inflammatory response, involving multiple organ system damages and alteration of the evaluation indicators, such as PLT count. Whereupon, the information of NEC patients without sepsis was analyzed using univariate regression, and 17 variables with *P *< 0.05 were considered as independent variables for stepwise multivariate non-conditional logistic regression analysis. The results showed that anemia, PLCR, and PCT were the final significant indicators in the model. Anemia (OR = 4.367, 95% CI: 1.853–10.291, *P* = 0.001) increased the risk of NEC in LBW neonates without sepsis, and increased PLCR (OR = 2.222, 95% CI: 1.633–3.023, *P *< 0.001) and PCT (OR = 1.368, 95% CI: 1.042–1.795, *P* = 0.024) could be the indicators in predicting the risk of NEC in LBW neonates without sepsis, as shown in Table 6.

**Table 6:** Multivariate logistic regression analysis of NEC occurrence in LBW neonates without sepsis.

	*B*	SE	*P*	OR	95% CI	
					Lower limit	Upper limit
Anemia	1.474	0.437	0.001	4.367	1.853	10.291
PLCR	0.798	0.157	<0.001	2.222	1.633	3.023
PCT	0.313	0.139	0.024	1.368	1.042	1.795

### Analysis of the effect of multiple factors and their correlation on NEC occurrence in neonates with sepsis

The information on NEC patients with sepsis was analyzed using univariate regression, and 10 variables with *P *< 0.05 were considered as independent variables for stepwise multivariate non-conditional logistic regression analysis. The results showed that only MPV was the final significant indicator entered into the model. Increased MPV (OR = 1.409, 95% CI: 1.017–1.953, *P* = 0.040) was the indicator to predict the risk of NEC in LBW neonates with sepsis (Table 7).

**Table 7:** Multivariate logistic regression analysis of NEC occurrence in LBW neonates with sepsis.

	*B*	SE	*P*	OR	95% CI	
					Lower limit	Upper limit
MPV	0.343	0.167	0.040	1.409	1.017	1.953

### PLCR and PCT in predicting NEC occurrence

**Figure 2:**
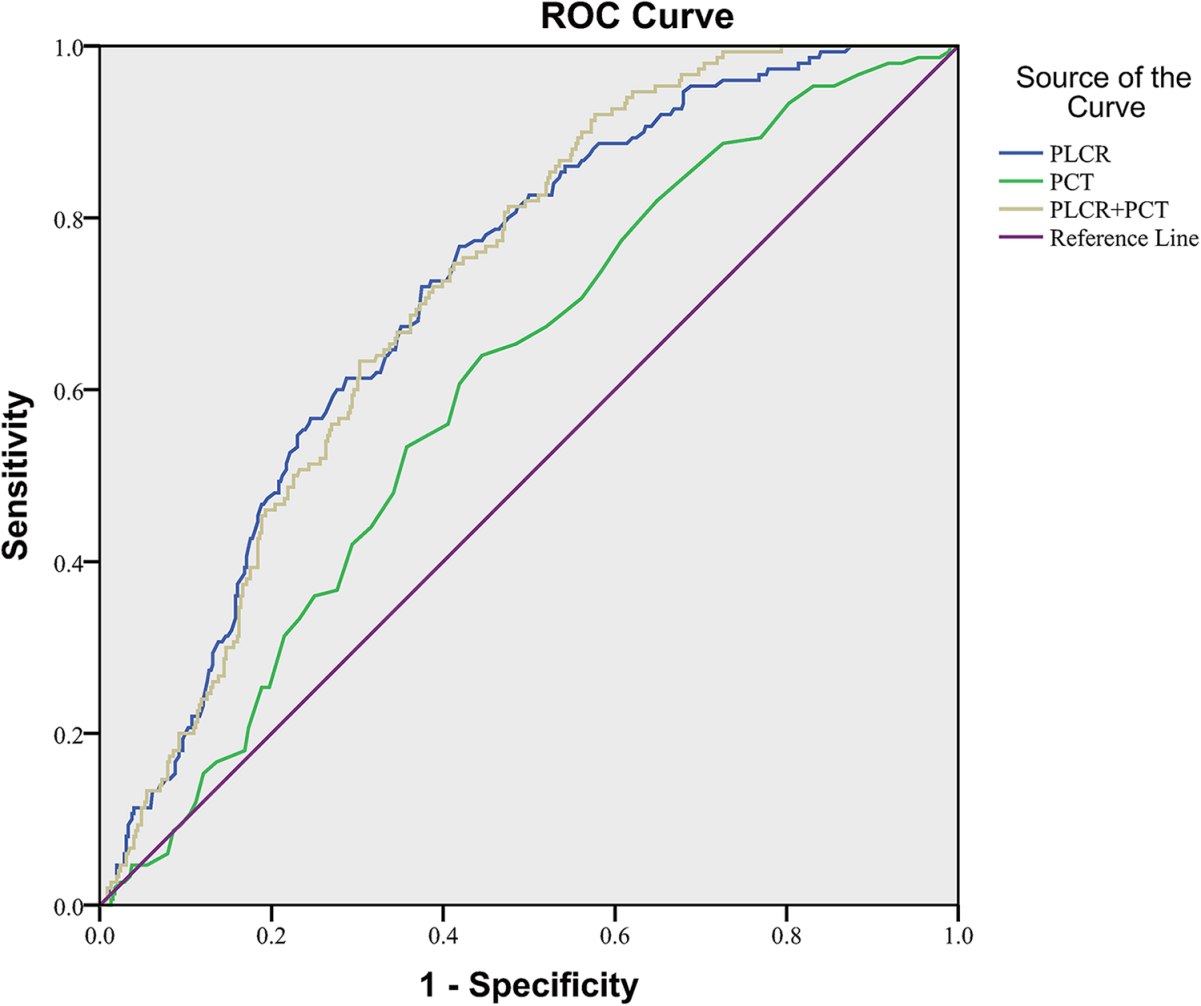
PLCR and PCT in NEC diagnosis of LBW neonates.

The ROC curve fitting analysis was used to evaluate the PLCR, PCT, and the combination of the two in NEC diagnosis. In all NEC patients in this study, the ROC curve area of PLCR diagnosis was 0.717 (*P* < 0.001), the sensitivity was 0.767, the specificity was 0.581, and the cutoff value was 33.55. The ROC curve area of PCT diagnosis was 0.606 (*P* < 0.001), the sensitivity was 0.640, the specificity was 0.560, and the cutoff value was 0.3350. The ROC curve area of PLCR–PCT diagnosis was 0.719 (*P* < 0.001), the sensitivity was 0.920, the specificity was 0.423, and the cutoff value was 0.1566 (Figure 2).

**Figure 3:**
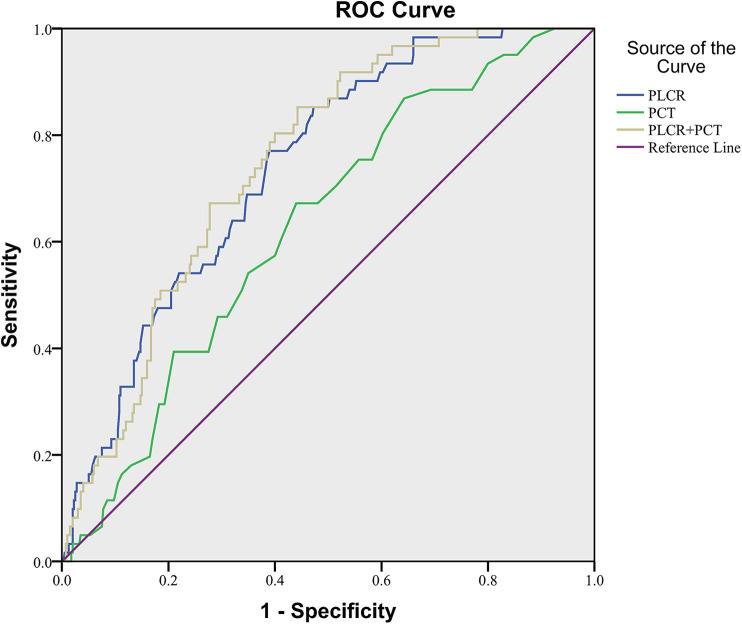
PLCR and PCT in NEC diagnosis of LBW neonates without sepsis.

In NEC patients without sepsis, the ROC curve area of PLCR diagnosis was 0.739 (*P *< 0.001), the sensitivity was 0.770, the specificity was 0.610, and the cutoff value was 33.55. The ROC curve area of PCT diagnosis was 0.629 (*P* = 0.001), the sensitivity was 0.672, the specificity was 0.560, and the cutoff value was 0.3350. The ROC curve area of PLCR–PCT diagnosis was 0.748 (*P *< 0.001), the sensitivity was 0.852, the specificity was 0.557, and the cutoff value was 0.1074. In conclusion, the value of the combination of PLCR and PCT and PCT alone in NEC diagnosis was not significantly higher than PLCR alone (Figure 3).

The values of these ROCs were acceptable.

## Discussion

The present study aimed to provide a pooled estimation of NEC in LBW neonates in China. The results showed that 2/100 LBW neonates developed NEC and that sepsis and anemia were the risk factors for NEC occurrence in LBW neonates. This might be the first study to show the superior value of PLT activation (especially PLCR), rather than PLT count, in predicting NEC occurrence in LBW neonates. Most studies postulate that neonatal sepsis is a major risk factor for NEC (10, 12, 27, 32). Currently, the correlation between anemia and NEC occurrence is under intensive focus, but the findings of such studies are yet controversial. The association between PDA and the occurrence of NEC due to hemodynamic changes is consistent with the findings of most studies (26). In this study, our results are suggestive that there is a correlation between anemia and NEC occurrence. The present study identified the risk factor for NEC occurrence in LBW neonates in NICUs in China and emphasized the value of PLT activation in NEC diagnosis of LBW neonates, thereby providing a new approach for future studies on NEC pathogenesis.

### Correlation between neonatal sepsis and NEC occurrence

Stratified analysis of sepsis identified the risk factors for NEC in LBW neonates. This study found that every 6/10 NEC patients presented sepsis and both EOS and LOS were risk factors for NEC, suggesting that the control of intrauterine infection and hospital infection is crucial in preventing NEC. In the event of sepsis infection, pathogen-produced toxins may directly damage the intestinal mucosa or activate immune cells to produce cytokines, thereby altering vascular permeability and tissue damage (3). These phenomena result in the accumulation of PLTs and white blood cells in capillaries, which in turn causes intestinal damage and eventually NEC by blocking blood flow, aggravating the intestinal mucosa, and initiating excessive multiplication of intestinal bacteria. Another study showed that sepsis increases the risk of NEC by threefold (27), which is similar to the current finding. Typically, neonatal sepsis induced by different types of microbes manifests as a variety of pathophysiology and can result in several complications and outcomes (32, 33). Therefore, neonatal sepsis is deemed a vital confounding factor in the etiological analysis of NEC. Importantly, we also found that the risk factors for NEC differed between neonates with and without sepsis. These patients were further grouped into NEC subjects with and without sepsis for analysis. The results showed that NEC occurrence was correlated with anemia, PLCR, and PCT in NEC subjects without sepsis under the interplay of multiple factors. However, only MPV was weakly correlated with NEC occurrence in NEC subjects with sepsis.

### Correlation between PLT activation indicators and NEC occurrence

PLT activation, rather than PLT count, was the earlier predictor for NEC occurrence. Pups with 2,4,6-trinitro-benzene sulfonic acid (TNBS)-mediated acute necrotizing ileocolitis had increased immature PLT fractions, high MPV, and increased megakaryocyte number/ploidy in the bone marrow, which are consistent with the clinical observations in human NEC (34, 35). These manifestations favored peripheral PLT consumption, but not decreased production, as the kinetic basis for thrombocytopenia (36). However, the literature showed that the PLT count decreased significantly in NEC patients compared to that in non-NEC patients and was correlated with the severity of NEC (37). Our results were different from the previous literature. The main reasons might be that the PLT in this study was counted 2 days before NEC diagnosis when NEC is in the early stage and the bone marrow is producing compensatory blood vigorously to maintain normal PLT count or increase PLT count. Additionally, PLCR and PCT were sensitive and increased at the early stage. Apparently, PLCR and PCT, not PLT count, could be used as indicators to predict the early risk in NEC patients without sepsis, indicating that PLT activation was the early predictor for NEC occurrence. Various types of sepsis affect the PLT through different pathways. PLT activation and depletion during NEC disrupt the mucosal wall established and lead to bacterial translocation across the damaged mucosa (18). PLTs repair bacterial damage to the vascular endothelium, resulting in increased platelet consumption, activated immune system, and enhanced PLT apoptosis (38). Other common viral infections, such as Torch or fungal infection, directly destroy the megakaryocytes or PLTs, inhibit bone marrow hematopoiesis, induce the production of autoantibodies to accelerate PLT destruction, and affect their levels in peripheral blood. Severe bacterial infections [such as GBS (group B streptococcus) infection] disrupt bone marrow hematopoiesis, leading to thrombocytopenia. Therefore, it could be speculated that the value of PLT indicators in predicting the occurrence of NEC in neonates with sepsis was not significant.

### Correlation between anemia and NEC occurrence

Anemia was a risk factor for NEC occurrence in patients without sepsis but not in those with sepsis. Previous studies on anemia promoting NEC occurrence yielded varied results. Patel et al. (13) and Singh et al. (26) speculated that anemia, rather than blood transfusion, was correlated with a high risk of NEC occurrence; however, other retrospective studies did not find such a significant effect (27, 39). Nonetheless, no stratified analysis of sepsis was performed in these studies. Herein, we proposed that anemia, rather than blood transfusion, increased the risk of NEC occurrence in patients without sepsis because anemia affects splanchnic perfusion and causes hypoxia, anaerobic metabolism, and accumulation of anaerobic metabolism products, such as lactic acids. These by-products disrupt the intestinal vascular regulation resulting in ischemic injury, thereby increasing the risk of NEC occurrence (40). Various microbes causing sepsis give rise to anemia, hypoxia, and intestinal tissue damage in the body and, hence, are deemed confounding factors that complicate the analysis of the results. In the NEC and sepsis subgroup, the lack of association between anemia and NEC development may be due to a series of severe lesions caused by sepsis that interfere with this factor; hence, more follow-up research is needed.

Nevertheless, the present study had some limitations. First, all the results were based on the largest women's hospital in Zhejiang Province (one of the largest women's hospitals in China), but the information recorded did not reflect NEC occurrence and the associated risk factors nationwide. Therefore, multicenter clinical studies are essential to further investigate the correlation between anemia, increased PLCR, increased PCT, and NEC occurrence in LBW neonates. Second, this case–control study had a retrospective design and, thus, was subject to information bias. Hence, a cohort study, as well as a randomized controlled clinical experiment, should be carried out in the future to substantiate the current findings.

## Conclusion

PLCR has a significant value in the early prediction of NEC incidence in LBW neonates without sepsis.

## Data Availability

The original contributions presented in the study are included in the article, further inquiries can be directed to the corresponding authors.
